# Tryptophan metabolism as a target in gut microbiota, ageing and kidney disease

**DOI:** 10.7150/ijbs.115359

**Published:** 2025-06-23

**Authors:** Hua Miao, Shui-Juan Zhang, Xin Wu, Ping Li, Ying-Yong Zhao

**Affiliations:** 1 School of Pharmaceutical Sciences, The First Affiliated Hospital of Zhejiang Chinese Medical University, Hangzhou, 310006, China.; 2 School of Pharmaceutical Sciences, Zhejiang Chinese Medical University, Hangzhou, 310053, China.; 3 Beijing Key Lab for Immune-Mediated Inflammatory Diseases, Institute of Clinical Medical Science, Department of Nephrology, China-Japan Friendship Hospital, Beijing, 100029, China.

**Keywords:** tryptophan metabolism, IDO1/2, aryl hydrocarbon receptor, gut microbiota, kidney disease, ageing

## Abstract

Aromatic amino acid tryptophan metabolism, particularly three main metabolism pathways including kynurenine, serotonin and indole-derived pathways are under the direct or indirect modulation of host-microbiota crosstalk in human physiology. Tryptophan metabolism is involved in the regulation of aging, immunity and intestinal homeostasis. Dysregulation of tryptophan metabolism ranging from bowel disease to kidney disease allow us to therapeutic targeting the tryptophan metabolism. This review summarizes recent advances in physiological and pathophysiological roles of tryptophan metabolism in health and disease such as ageing-related disease, bowel disease and renal disease. Decoding the sophisticated imbalance between tryptophan metabolism pathways will expedite a comprehensive understanding of the pathogenesis of human diseases and highlight the opportunities and challenges for medication research and development in multiple diseases. This review presents concept-driven diagnostic and therapeutic strategies for the management of patients with kidney disease by gut-kidney-aging axes.

## 1. Introduction

**Figure 1:**
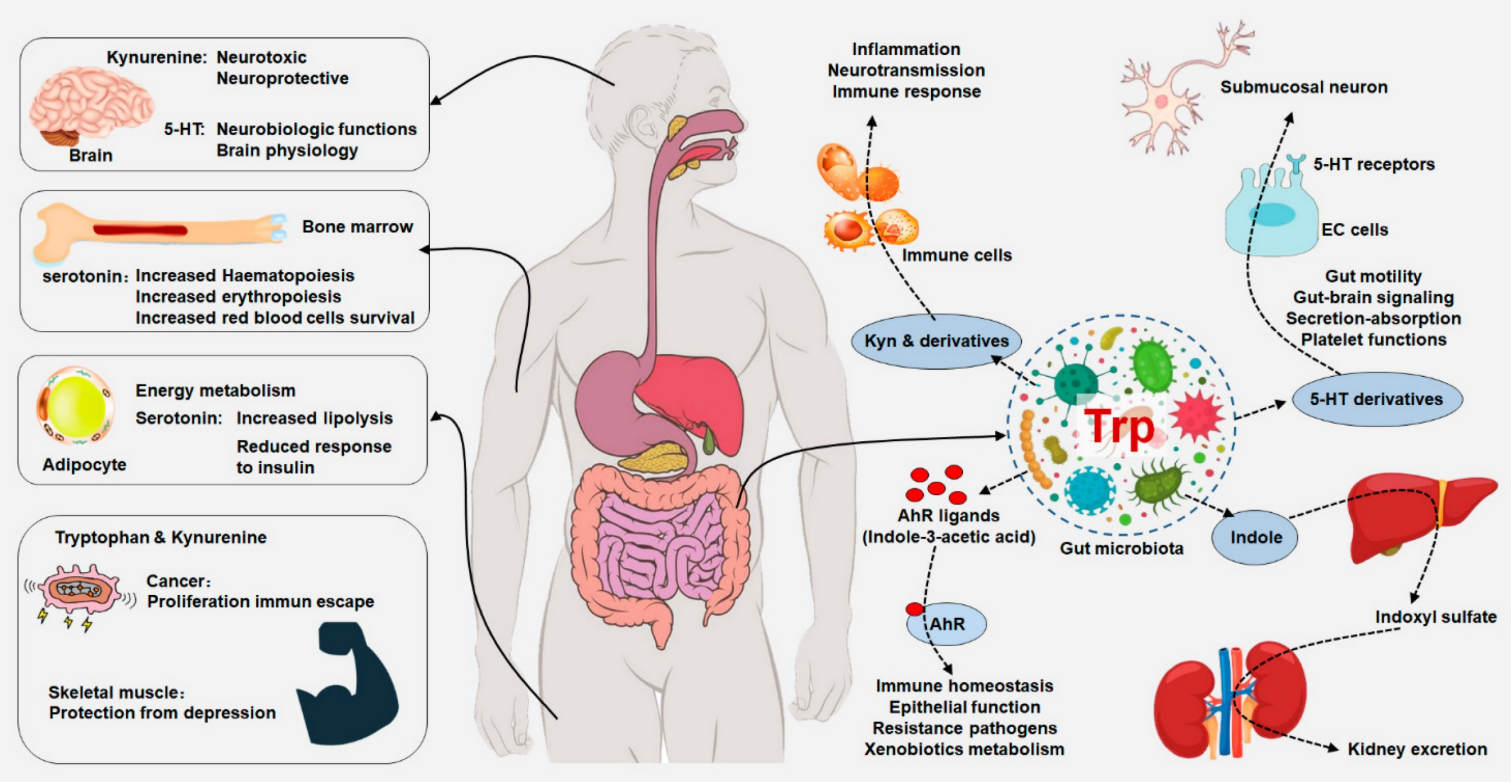
**Integrated tryptophan metabolism regulates host physiology and gut microbiota.** Dietary tryptophan can be metabolized by host and gut microorganism that could modulate local and distant host physiological functions, including immune homeostasis and barrier physiology. Tryptophan metabolism plays an important role in neurobiological functions, immune responses and inflammatory mechanisms. Peripheral production of serotonin by enterochromaffin cells is also under the influence of the gut microorganism. Gut microbiota-derived tryptophan metabolite serotonin has a variety of local effects. Tryptophan is converted by gut microorganism into AHR ligands that regulated AHR signaling that maintained immune homeostasis, epithelial function, resistance to pathogens and xenobiotics metabolism. Gut microbiota has an indirectly effect on central serotoninergic pathways by regulating tryptophan and tryptamine availability.

Tryptophan is an essential amino acid for humans, which the human organism cannot synthesize and must be supplied by exclusively dietary protein 1, 2. Tryptophan and its metabolites regulate a variety of important physiological and pathological processes 1, 3 (Figure 1 and Table 1). In addition to protein synthesis, it contributed to multiple biological processes, including the production of biogenic amines such as serotonin, melatonin and tryptamine as well as a variety of tryptophan metabolites from kynurenine pathway 4, 5. For example, kynurenic acid regulated adipose tissue energy homeostasis and influenced on systemic energy expenditure and inflammation via activation of orphan G protein coupled receptor 35 6. Tryptophan metabolism is involved in multiple physiological processes such as ageing, immunological effect, neuronal function and environmental interfaces 1, 7, 8. In mammals and yeast, tryptophan contributed to the synthesis of nicotinamide adenine dinucleotide (NAD^+^), an important coenzyme used for energy metabolism 9. Taken together, these functions suggest that tryptophan metabolism becomes important part of the cellular and organismal communication (Figure 1). Increasing evidence has suggested that the dysregulation of tryptophan metabolism was implicated in inflammatory bowel disease (IBD), irritable bowel syndrome (IBS) and kidney diseases and tryptophan metabolism is a promising therapeutic target for the treatment of various diseases 10-12. Here, we review the recent insights regarding the role of the tryptophan metabolism in physiology and ageing implicated in crosstalk between different tissues (host-microbial crosstalk) with a focus on the consequences on a wide range of important diseases such as ageing, bowel and kidney diseases. Therapeutic progress in targeting tryptophan metabolism was also highlighted. We further discussed future efforts to explore the potential role of tryptophan metabolism as a hub linking gut-kidney-aging axes.

## 2. Tryptophan Metabolism: Pathways and Physiology

**Figure 2:**
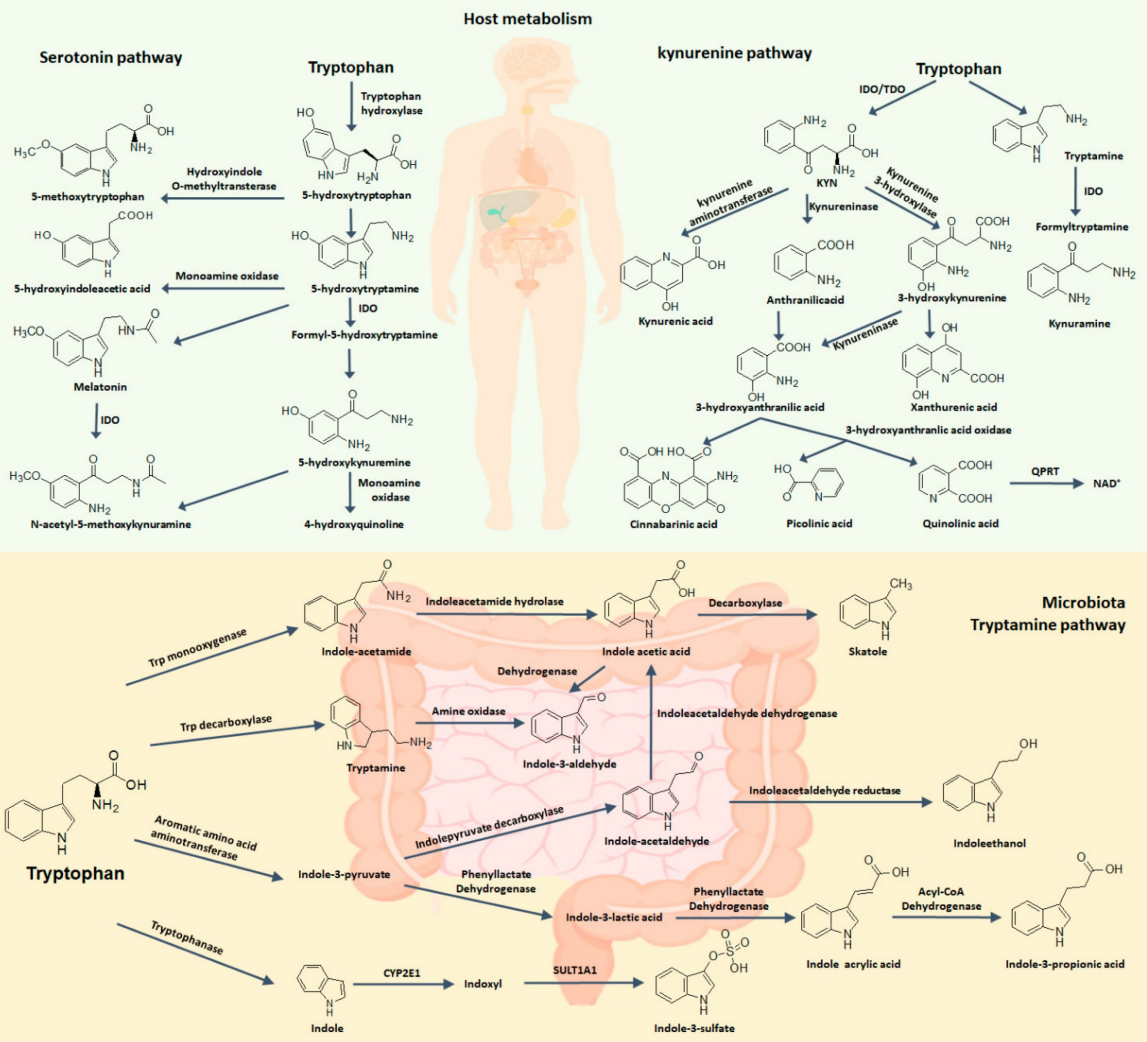
** A summary of the pathways of tryptophan metabolism.** Serotonin, kynurenine, and indole pathways are three mainly metabolic pathways of tryptophan metabolism. A small fraction of free L-tryptophan is used for protein synthesis and the production of neurotransmitters such as serotonin and neuromodulators such as tryptamine. However, over 95% of free tryptophan is a substrate for the kynurenine pathway of tryptophan degradation, which generates several metabolites. Tryptophan is converted into various metabolites by the gut microorganism such as indole, tryptamine, ILA, indoleacrylic acid, IAld, IAA, IPA, indole-3-pyruvate and indole-3-acetaldehyde and may affect host physiology in numerous ways.

Free tryptophan levels in the body are determined by dietary protein. Among essential amino acids, L-tryptophan is the least abundant, with plasma concentrations of approximately 40-80 μM in humans and approximately 60-100 μM in mice. In humans, related-enzymes and metabolites of tryptophan metabolism are localized in different cells and tissues, where their expression is tightly modulated 13. However, the dysregulation of tryptophan and its metabolites have been involved in a wide range of pathologies (Table 1). The linkage of tryptophan metabolites to a range of diseases has led to substantial efforts to target tryptophan metabolism pathway, particularly via inhibition of several key enzymes 14. Tryptophan is metabolized by three main pathways including kynurenine pathway and 5-hydroxytryptamine (5-HT) pathway in host cells as well as indole pathway (also called tryptamine pathway) in gut microbiota (Figure 2).

### 2.1. Kynurenine pathway

After absorption by host cells, approximately 95% of free L-tryptophan as a substrate is metabolised via the kynurenine pathway, which generates a number of metabolites with distinct bioactivities in physiology and disease status 1, 4, 5. Free tryptophan is degraded into kynurenine via two cytosolic and rate-limiting enzymes including tryptophan 2,3-dioxygenase (TDO: EC 1.13.11.11) and indoleamine 2,3-dioxygenase (IDO: EC 1.13.11.17) that are intracellular non-secreted haem enzyme that cleave the 2,3-double bond of the aromatic indole ring of tryptophan via the incorporation of molecular oxygen, produce N-formylkynurenine, which is converted to L-kynurenine via deformylation (Figure 2). Monomeric TDO expression is highly conserved across different species including both eukaryotes and prokaryotes, but homotetrameric IDO is only confined in eukaryotes. IDO included two high amino acid homologies, but distinct enzymes encoded by two different genes, namely IDO1, IDO2. The genes encoding IDO1 and IDO2 are adjacent on chromosome 8, indicating their common origin 15. IDO1 and IDO2 have 43% amino acid identity, containing residues critical for catalytic activity.

Already in 1967, IDO1 was isolated and identified from rabbit intestine 16. The two enzymes can both convert the same substrates, such as tryptophan, tryptamine and 5-hydroxytyptamine whereas IDO1 has higher catalytic activity 17. The previous study indicated that Michaelis Constant of IDO2 is 100-fold higher than physiological tryptophan levels which produces the direct role of IDO2 in tryptophan degradation and suggests another, so far unidentified natural substrate for the enzyme 18. The IDO1 expression is ubiquitous, while IDO2 expression is only presented in brain, liver, spleen, lung, kidney, thymus, placenta, colon and small intestine 19. TDO affects systemic tryptophan concentrations by controlling tryptophan concentrations in the blood, while IDO acts locally to regulate tryptophan concentrations in response to inflammation 20. The two enzymes are not constitutively expressed in most cells but can be induced by different inflammatory stimuli such as lipopolysaccharides, cytokines and pathogens 21. Among proinflammatory cytokines, interferon-γ is one of the elementary mediators for IDO1 expression, yet it seems to only slightly affect IDO2 expression 22. The two enzymes are activated in epithelial cells, endothelial cells, polymorphonuclear cells, eosinophils and fibroblasts by pro-inflammatory cytokines during infection or inflammation. Other cytokines, such as tumor necrosis factor-α, interleukin-2 and interleukin-1β, could enhance the interferon-γ-induced IDO1 expression. On the other hand, several antiinflammatory cytokines including transforming growth factor-β, interleukin-4 and interleukin-10 were demonstrated to inhibit IDO1 expression by interferon-γ 23. Additionally, a variety of signaling pathways including aryl hydrocarbon receptor (AHR), transforming growth factor-β receptor, tumor necrosis factor receptor, Toll-like receptors, interferon-γ receptor and interferon-β receptor are demonstrated to induce or maintain IDO1 expression 15. The promoter of IDO1 gene includes some nucleotide sequences, such as non-canonical nuclear factor kappa B consensus sequences, interferon sequence response-like elements and palindromic γ-activated sequences that modulate gene expression. IDO1 transcriptional expression can be enhanced by several transcription factors, such as interferon regulatory factor 8 and forkhead box O3, whereas they are inhibited via DNAX activation protein 24. By contrast, IDO2 expression was demonstrated to be primarily induced by interferon regulatory factor 7. Regarding post-transcriptional regulation, two immunoreceptor tyrosine-based inhibitory motifs are known to suppress the cytokine signaling 3-dependent proteasomal degradation of IDO1 protein in the presence of interleukin-6 25. Taken together, these enzymes are promising therapeutic targets. Compared with monomeric IDO1, the homotetrameric TDO has a less substrate range. In mammals, TDO expression is induced by L-tryptophan and glucocorticoids in several tissues. Notably, TDO is enantiomer-specific and can only cleave L-tryptophan. TDO is a critical regulatory enzyme in modulating circulating L-tryptophan levels and is regarded to have an important role in supplying NAD^+^ via kynurenine pathway.

The activities of these enzymes lead to the accumulated metabolites, chiefly kynurenine. Kynurenine may be converted to anthranilic acid by kynureninase and kynurenic acid by kynurenine aminotransferases, the latter step being important for controlling the production of neuroprotective kynurenic acid 26. Especially in brain, kynurenine may be transaminated into kynurenic acid through mitochondrial aspartate aminotransferase (encoded by GOT2) 7. Independently, kynurenine monooxygenase can convert kynurenine into neuroactive and neurotoxic metabolites including quinolinic acid. Quinolinic acid can be converted to a coenzyme NAD^+^ used for energy metabolism in certain cell types, but the physiological function of this de novo production of NAD^+^ by kynurenine pathway remains enigmatic as NAD^+^ is produced primarily by salvage 27.

### 2.2. Serotonin pathway

Tryptophan can produce several important metabolites such as 5-hydroxytryptophan (5-HTP), serotonin and 5-methoxytryptophan (5-MTP) via several important enzymes including tryptophan hydroxylase 1 (TPH1), tryptophan hydroxylase 2 (TPH2) and hydroxyindole-O-methyltransferase (HIOMT) in serotonin pathway 28 (Figure 2). Approximately 1-2% of ingested free tryptophan follows serotonin pathway and can convert in neurotransmitters such as serotonin through tryptophan hydroxylase and aromatic amino acid decarboxylase. 5-HTP is a key intermediate metabolite of serotonin biosynthesis and melatonin biosynthesis. In neuronal cells, L-tryptophan could be converted to 5-HTP by TPH2 and then 5-HTP is converted to 5-HT by aromatic amino acid decarboxylase 29. However, more than 90% 5-HT in the body is produced in gut and especially in enterochromaffin cells, a specialized subtype of intestinal epithelial cell. This process occurs via TPH1 that produces 5-HTP and 5-HTP is further metabolized into 5-HT. Under physiological conditions, peripheral 5-HT does not cross the blood-brain barrier. Peripheral 5-HT exhibits many functions in the gastrointestinal tract and is associated with a largely variety of human physiological functions by activating specific 5-HT receptor 30 (Figure 1). Specifically, 5-HT, as an important gastrointestinal signaling molecule, can express signals from the gut to intrinsic or extrinsic neurons and affect intestinal peristalsis and motility, vasodilatation and nutrient absorption. Moreover, the serotonin-selective reuptake transporter (SERT; encoded by SLC6A4 gene), expressed in the apical and basolateral membrane of intestinal epithelial cells, acts as a sponge to remove 5-HT from the interstitial space after production by enterochromaffin cells. This key molecule implicated in the local regulation of 5-HT availability is also responsible for 5-HT reuptake in the brain.

5-HT can convert into melatonin through two-step enzymatic conversion reactions. 5-HT is further converted to N-acetyl-5-hydroxytryptamine (N-acetyl-5-HT) via arylal kylamine N-acetyltransferase (AA-NAT) and N-acetyl-5-HT is converted to melatonin (N-acetyl-5-methoxytryptamine) via HIOMT in pineal cells 31. Melatonin is converted to indole derivatives through three major pathways. Melatonin is degraded through cytochrome p450s including CYP1A2, CYP1A1 and CYP1B1 to 6-hydroxy-melatonin which is converted to 6-sulfatoxy-melatonin via sulfotransferase; Melatonin converts to N^1^ acetyl-N^2^ formyl 5-methoxykynuramine by myeloperoxidase or IDO which is further converted to N^1^ acetyl-5-methoxykynuramine by formamidases; Melatonin is deacetylated to form 5-methoxytryptamine (5-MT) 32.

As little is known about 5-MTP production in mammalian cells, 5-MTP production by fibroblasts must be validated. L-tryptophan is converted to 5-HTP via TPH1 and 5-HTP is converted to 5-MTP via HIOMT in fibroblasts 33, 34.

### 2.3. Indole pathway in the gut

The diverse and dynamic microbiome of the human gastrointestinal tract played a critical role in health and host nutrition 10. A mutualistic relationship between host and gut microbiota are closely associated with complex molecular crosstalk, which is fundamental for intestinal homeostasis 5. A large array of metabolites mediated the crosstalk between host and its microbiome 3, 10. Currently, the three most common categories of metabolites involved in host-microbiota interactions are short-chain fatty acids produced via microbiome from fiber fermentation; bile acids produced in the liver and transformed via microbiome before re-affecting the host; and tryptophan metabolites 35, 36, which are the topic of this review.

A number of bacterial species have been demonstrated to convert tryptophan into indole and indole derivatives such as tryptamine, indole-3-propionic acid (IPA), indole-3-lactic acid (ILA), indoleacrylic acid, indole-3-aldehyde (IAld), indole-3-acetic acid (IAA), indole-3-pyruvate and indole-3-acetaldehyde, which can all affect host physiology in multiple pathways (Figure 1 and Table 1). The earliest study demonstrated that tryptophan could convert into indole by *Escherichia coli* and* Vibrio cholerae* 37. Later, indole production has been used as a diagnostic biomarker to distinction *E. coli* from other enteric bacteria. Because indole has been found for 100 years ago, many indole-producing bacterial species have been found and have been well covered in a previous review 38, 39. Briefly, indole is produced through the action of the enzyme tryptophanase (TnaA; EC4.1.99.1) that is expressed in numerous Gram-negative and Gram-positive bacterial species including *E. coli, Bacteroides* spp. and* Clostridium* spp. It has been long thought that gut microbiota synthesize tryptophan from indole as a carbon source via TnaA. However, reaction equilibrium is largely likely to indole production from tryptophan 38, 39. Recent several findings have demonstrated that microbial species can produce various tryptophan metabolites through several other metabolic pathways. For instance, *Clostridium sporogenes* can convert tryptophan into tryptamine, IPA and ILA 40. Similarly, *P. anaerobius, P*. *russellii* and *P. stomatis belonged to Peptostreptococcus* spp. can convert tryptophan to IPA and indoleacrylic acid, possibly owing to the presence of the phenyllactate dehydratase gene cluster (*fldAIBC*) on the chromosome in these species 41. Indeed, a homologue of this cluster is demonstrated to be able to convert tryptophan into ILA and IPA in *C. sporogenes*. Moreover, homologue gene-clusters were demonstrated in* Clostridium botulinum*, *Clostridium cadaveris* and *Peptostreptococcus anaerobius* in line with their capability to produce IPA. Lactobacilli are another group of bacteria capable of converting tryptophan. *Lactobacillus* spp. can convert tryptophan to ILA and IAld through indolelactic acid dehydrogenase (ILDH) and aromatic amino acid aminotransferase (ArAT) 42. *Ruminococcus gnavus* can convert tryptophan into tryptamine via tryptophan decarboxylase enzyme. Several *Bacteroides* species, and *Clostridium bartlettii* have been demonstrated to produce IAA and ILA whereas *Bifidobacterium* spp. has been demonstrated to produce ILA. Finally, the common intestinal metabolite 3-methylindole (skatole) that has been extensively demonstrated as the cause of off-flavor in pork is generated by decarboxylation of IAA by* Clostridium* spp. and *Bacteroides* spp 43. In addition, indole can be further metabolized into indoxyl sulphate, a cometabolite generated from indole in the liver by cytochrome P450 enzymes, such as CYP2E1 and sulfotransferase. A growing body of publications suggests that microbial-derived tryptophan metabolites are important signaling molecules in microbial communities and in host-microbial crosstalk, and may contribute to intestinal and systemic homeostasis.

## 3. Tryptophan Metabolites in Diseases

### 3.1. Tryptophan metabolism in ageing-associated diseases

Ageing is an important risk factor for many diseases, such as cancer, neurodegenerative disorders and kidney diseases 44-46. Several previous publications have demonstrated that tryptophan metabolism played an important in ageing and age-related diseases in various model organisms, such as worms, flies, mice, rat and yeast 47. Transcription factor AHR is a cytoplasmic receptor that is activated by a variety of tryptophan metabolites (Figure 1). Increasing studies have demonstrated that activated AHR signalling by tryptophan metabolites, such as indole-3-carboxyaldehyde, IAld, indoxyl sulphate, 5-hydroxyindole-3-acetic acid and cinnabarinic acid, was implicated in ageing-related pathogenesis and could be considered as a therapeutic target in ageing-related tissue fibrosis 48.

Kynureninase was demonstrated as one of the most differentially expressed genes in age-related changed gene expression in the peripheral blood of adult individuals 49. Knockdown of kynureninase by shRNA prolonged lifespan than that realized with knockdown of any of other differentially expressed genes in *C. elegans*, indicating a key contribution of kynureninase to ageing 49. As NAD^+^ is emerging as a potential lifespan-extending molecule, NAD^+^ possibly exerts an extension lifespan effect in kynurenine pathway. The longer lifespan in invertebrates is a consequence of reduced activity of kynurenine pathway, while prolonged lifespan by external dietary supplement of other NAD^+^ precursors would argue that an increased activity of kynurenine pathway would also be beneficial. Further study will be required to reveal these contradictory conclusions.

Tryptophan levels in rat's liver, kidney and brains were decreased with age while kynurenine levels were increased in these tissues 50. A study across 26 mammalian species demonstrated that kynurenine/tryptophan ratio in liver of healthy adult animals was related to species-specific maximum lifespan 51; species that exhibited a higher kynurenine/tryptophan ratio were shorter lived 50. Two independent publications have also demonstrated that kynurenine/tryptophan ratio that reflected tryptophan decomposed rate was significantly increased in old age people, indicating that ageing was paralleled by the accelerated tryptophan degradation in kynurenine pathway 52, 53. One of these studies demonstrated that a higher kynurenine/tryptophan ratio at the start of the study period predicted higher mortality in an individual group in their nineties 52. Recently, inhibition of several important enzymes of kynurenine pathway has presented direct evidence for a key role of tryptophan metabolism in age-related physiopathology 8. *In vitro* experiment demonstrated that rapamycin treatment suppressed IDO activity in blood cells, indicating a relationship between tryptophan metabolism and target of rapamycin pathway 54. It will be interesting to learn whether inhibition of the first step in tryptophan degradation regulates lifespan in higher organisms. The latest study suggested that IDO-kynurenine pathway induced NOD-like receptor protein 3 inflammasome activation-mediated postoperative cognitive impairment in aged mice, whereas, treatment with IDO inhibitor 1-DL-methyl-tryptophan decreased the levels of kynurenine and kynurenic acid, increased tryptophan levels and improved learning and memory abilities 55. The recent study showed that the IDO inhibitor 1-DL-methyl-tryptophan attenuated DNA damage response, reduced p21, p16, and senescence-associated β-galactosidase activities, restored cell proliferation, and reduced interleukin-6 production while AHR inhibitor CH223191 did not affect these results in renal tubular epithelial cells senescence under anoxia or reoxygenation 56. As TDO inhibitors are available and TDO knockout mice are viable, these models could reveal TDO inhibition effects on lifespan. However, the inhibition of tryptophan metabolism could exacerbate immune responses upon inflammatory stimulation and might result in a deteriorated inflammation milieu, which could have severe consequences on health 50. Taken together, these findings indicated a causal connection between ageing and kynurenine pathway.

### 3.2. IBD and IBS

During the last five years, a growing body of literature suggests that IBD and IBS have become one of the high risk and morbidity directly associated with gut microbiota and host metabolism 57, 58. Mounting studies have demonstrated serum tryptophan levels were significantly decreased in a myriad of diseases including IBD and IBS. Several seminal studies have illuminated dysregulation of tryptophan metabolism with linked to intestinal microorganisms 15, 59. The decrease in gut microbiota-derived tryptophan metabolites as AHR ligands in IBD patients was influenced by genetic factors 59. Serum tryptophan levels are lower in patients with IBD than in healthy controls. Compared with patients with ulcerative colitis, serum tryptophan levels were also decreased in patients with Crohn's disease, a chronic inflammatory disorder of the gastrointestinal tract 60. In addition, plasma tryptophan levels were demonstrated to be reduced in Crohn's disease 61, 62, whereas faecal tryptophan levels are increased compared with healthy controls 63. These observations suggest that altered tryptophan metabolism are implicated in the pathological process of IBD. Importantly, not only tryptophan appears to exert a critical role in the pathogenesis of IBD. Depletion of intestinal tryptophan metabolites also led to exacerbating IBD, as previous study revealed that IBD patients have reduced faecal levels of the IAA 59, which was accompanied by downregulated AHR expression in the intestinal tissue of IBD patients 64 and activated AHR protected humanized mice against colitis by inducting regulatory T cells 65. In addition, serum IPA levels were significantly decreased in patients with active colitis compared with healthy controls 66, and oral administration of indole, and IPA is demonstrated to alleviate colonic inflammation in mice 67. Both gut local and systemic IDO1 overexpression in body were supported by significantly increased IDO1 activity in active IBD patients and by negative association between serum tryptophan levels and C-reactive protein expression compared with non-active IBD patients 60. Taken together, these diverse examples suggest that alterations in tryptophan metabolism involved in IBD and might have an important active role in disease pathogenesis. The production of AHR agonists might also indicate the exacerbated IDO activation that has a direct effect on the gut microbiota under physiological conditions (Figure 1).

Although the pathogenesis of IBS is not completely clear, a number of studies have demonstrated that the dysregulation of tryptophan metabolism contributed to the etiology of IBS 68. For example, increased serum kynurenine levels might be associated with peripheral IDO1 activity that was positively correlated with IBS severity 69. Alterations in gut motility are linked to the aberrant metabolism of 5-HT in IBS. The expression levels of TPH1 and serotonin-selective reuptake transporter reduced in rectal biopsies of IBS patients compared with healthy controls 70. In contrast, 5-HT levels in colon are decreased and increased in constipation- and diarrhea-predominant IBS, respectively 69. In addition, the study has demonstrated that the effects of the gut microbial on 5-HT production in mice, suggesting that microbiota-mediated dysregulation of 5-HT production are partly implicated in IBS 71. A variety of 5-HT receptors mediated the multiple effects of 5-HT that could evoke specific functions in specific organs. 5-HT receptor subtypes including 5-HT_3_ and 5-HT_4_ mostly expressed in the gastrointestinal tract linked 5-HT to visceral motility disorders. 5-HT has been exploited as an intervention target with the use of 5-HT_3_ receptor antagonists and 5-HT_4_ receptor agonists presented beneficial effects in the diarrhea- and constipation-related IBS, respectively. Taken together, targeting tryptophan metabolism by modulating the endogenous gut microbiota may provide an alternative therapeutic strategy for prevention and treatment of IBD.

### 3.3. Tryptophan metabolites in kidney diseases

Kidney disease is an increasingly public health issue associated with high morbidity and mortality 72-74. High-throughput metabolomics has advanced new biomarker identification and promoted the understanding of the biochemical mechanism of kidney disease 75-78. Based on metabolomic technique, a growing body of literature suggests that the dysregulation of various metabolites particularly tryptophan-derived metabolites was implicated in kidney diseases particularly chronic kidney disease (CKD) 3, 79, 80. Previously, our preliminary study has demonstrated the decreased plasma 5-MTP levels in patients with end-stage renal disease (ESRD) and its levels positively correlated with estimated glomerular filtration rate (eGFR) 81. Early detection and accurate monitoring of CKD could improve care and retard from CKD to ESRD. The latest study identified five metabolites including 5-MTP and their levels correlated with clinical biomarkers of CKD by metabolomics in 2155 participants including patients with stage 1-5 CKD and healthy controls 33. 5-MTP levels were significantly decreased with progressive CKD and in mouse kidneys after unilateral ureteral obstruction (UUO). Extensive studies have demonstrated that inhibitor of kappa B (IκB)/nuclear factor kappa B (NF-κB) signaling pathway, and kelch-like ECH-associated protein 1 (Keap1)/nuclear factor erythroid 2-related factor 2 (Nrf2) signaling pathway played a central role in CKD 82-84. Treatment with 5-MTP alleviated renal fibrosis, suppressed IκB/NF-κB signaling pathway, and improved Keap1/Nrf2 signaling pathway in mice with UUO or ischemia/reperfusion injury and in human kidney cells 33. Overexpression of TPH-1 ameliorated renal damage by retarding renal inflammation and fibrosis, whereas TPH-1 deficiency aggravated renal damage and fibrosis by activation of NF-κB and inhibition of Nrf2 pathways 33. These results indicated that TPH-1 may serve as a target in the treatment of CKD. Except for 5-MTP, our previous studies have also demonstrated the decreased tryptophan levels and increased levels of tryptophan metabolites including kynurenine, indoxyl sulfate (IS), 4-aminohippuric acid and hippuric acid in plasma while the increased tryptophan levels and the levels of decreased four metabolites in urine from ESRD patients 81. In addition, it has been reported that the dysregulation of tryptophan and its metabolites were observed in adults without diabetes in the fasted state including patients with CKD and patients with normal eGFR who underwent hyperinsulinemic-euglycemic clamp 85. Moreover, the dysregulation of serum IPA and hippuric acid were demonstrated in dietary acid load of adults with CKD 86. Adenine treatment led to the increased levels of IS and hippuric acid in kidney tissues of rats 3. Moreover, the dysregulation of kynurenic acid and hippuric acid were demonstrated in the plasma and urine of mice with type 1 diabetes and diabetic nephropathy 87. These findings demonstrated dysregulation of tryptophan and its metabolites were implicated in CKD.

Increasing publications have showed that the dysbiosis of gut microbiota was related to progressive CKD and its complications 88-92. Our study showed that kidney function decline was related to decreased *Lactobacillus* and *Bifidobacterium* and changed tryptophan-derived indole metabolites in cationic bovine serum albumin-induced membranous nephropathy rats 93. Our study further demonstrated that deceased *Lactobacillus johnsonii*, *L. reuteri*,* L. vaginali*s, *L. murinus* and *Bifidobacterium animalis* positively correlated with deceased IAld, indole-3-pyruvic acid and tryptamine, and negatively correlated with elevated IAA and ILA in membranous nephropathy rats 93. The changes in probiotics and tryptophan-derived indole metabolites were also demonstrated in idiopathic membranous nephropathy patients. Further data showed that membranous nephropathy rats were related to AHR pathway 82, 93. Our latest study identified taxonomic chain Bacilli-Lactobacillales-Lactobacillaceae-*Lactobacillus*-*Lactobacillus johnsonii* correlated with progressive CKD in patients, whose abundance correlated with serum creatinine levels 3. The relative abundance of* L. johnsonii* decreased with progressive CKD in adenine-induced rats.* L. johnsonii* supplementation attenuated renal injury 3. Serum IAld levels that strongly negatively correlated with serum creatinine levels in CKD rats, was significantly decreased in rats induced by UUO and 5/6 nephrectomy as well as CKD patients 3. IAld treatment retarded renal damage by inhibiting AHR pathway in rats with CKD or UUO, and 1-hydroxypyrene-mediated HK-2 cells 3. The IAld beneficial effect was partially abrogated in AHR deficiency mice and HK-2 cells. Furthermore, our result showed that *L. johnsonii* treatment attenuated renal damage through blocking AHR pathway via elevated IAld levels 3. These data presented a profound understanding of how microbial-derived tryptophan metabolism influences host and finds potential mechanism for therapeutic intervention for CKD.

Acute kidney injury (AKI) is a syndrome characterized by sudden declining renal excretory function, with a consequent failure in maintaining fluid, acid-base and electrolyte balance 94, 95. Recent study demonstrated increasing IS and hippuric acid levels in the cerebrospinal fluid in AKI 96. Extensive studies have suggested that even apparent complete recovery from AKI is associated with a subsequent risk for CKD development 97-99. Incomplete recovery from a severe episode of AKI is recognized as one of important pathways to CKD development, and the progression from CKD to ESRD 100, 101. CKD is a highly incidence and prevalence public health problem all over the world and ultimately progress to renal failure 102-104. Many AKI patients finally progress to CKD 105-107. Decreased levels of kynurenine and hippuric acid in urine were observed the transition of AKI-to-CKD rats induced by folic acid 108. Melatonin treatment could retard renal fibrosis through inhibition of interaction of Smad3 and β-catenin pathway and regulation of Gas6/Axl-NF-κB/Nrf2 axis in AKI-to-CKD continuum 109. These findings demonstrated dysregulation of tryptophan and its metabolites were involved in AKI-to-CKD continuum.

Dysbiosis in composition and structure of the gut microbiome community resulted in dysregulation of endogenous metabolites in various diseases including kidney diseases 110-114. Serum indoxyl sulphate levels were associated with increased inflammatory biomarkers in stage 3-4 CKD patients, such as glutathione peroxidase and interleukin-6 115. Our latest review summarized the pathogenic association between gut microbiota and metabolites particularly in kidney diseases covering CKD, IgA nephropathy, nephrolithiasis, hypertension, acute kidney injury, hemodialysis and peritoneal dialysis 116. We further employed 16 rRNA sequence and untargeted metabolomic analyses to reveal the changes in colonic microbiota and plasma metabolites and their relationship with renal fibrosis by using UUO and 5/6 nephrectomized rat models 117, 118. Tryptophan and its metabolites including kynurenine, 5-HTP and 5-HT levels which were linked with renal fibrosis correlated with nine specific genera 117. Plasma tryptophan levels positively correlated with the levels of* Turicibacter*,* Clostridium IV*, *Pseudomonas* and *Lactobacillales* and negatively correlated with the levels of* Blautia*,* Oscillibacter* and *Intestinimonas* which contained the genes encoding tryptophan synthase (K16187), IDO (K00463) and TDO (K00453) and their corresponding enzymes (EC:1.13.11.52 and EC:1.13.11.11) that aggravated renal fibrosis 117. In 5/6 nephrectomized rats, decreased tryptophan levels positively correlated with *Clostridium IV* and negatively correlated with* Blautia, Enterorhabdus, Allobaculum*,* Clostridium sensu stricto* and* Escherichia shigella*, while the increased plasma levels of hippuric acid and kynurenic acid positively correlated with *Enterorhabdus*, *Parasutterella, Blautia, Clostridium sensu stricto* and* Escherichia shigella* 118. These findings demonstrated that renal fibrosis resulted in profound changes in gut microbiome and circulating tryptophan-derived metabolites, events that contribute to the pathogenesis of inflammation and renal fibrosis.

AHR is activated by a largely range of structurally diverse compounds from the environment, natural products, microbiome and host metabolism 119-122. Extensive studies have suggested that tryptophan-derived IS and IAA were recognized as the endogenous AHR ligands and triggered AHR activation 123, 124 (Figure 1). A study reported that indole metabolites upregulated tissue factor expression by an AHR-dependent pathway in stages 3-5D of CKD patients. Upregulated tissue factor expression was positively correlated with the levels of serum IS and IAA in patients with CKD 125. IS and IAA further upregulated the expression of eight AHR downstream genes including *CYP1A1*, *CYP1B1* and *CYP1A2* in human umbilical vein endothelial cells 125. Another study revealed that IAA activated AHR/p38MAPK/NF-κB pathway, which mediated cyclooxygenase-2 (COX-2) expression, and IAA elevated the production of reactive oxygen species both *in vivo* and *in vitro* 126. In addition, the IS levels significantly correlated with AHR activities in patients with ESRD 127. Moreover, monocytes respond to IS through AHR signalling and consequently upregulate tumour necrosis factor alpha expression in ESRD patients 128. These findings demonstrated that tryptophan metabolites mediated renal fibrosis by AHR activation (Figure 1). Taken together, targeting microbial-tryptophan metabolic pathway improved patients with CKD. Therefore, indole pathway is the most promising for CKD intervention.

## 4. Concluding Remarks and Future Perspectives

In summary, this review presented tryptophan metabolism as a hub linking gut-kidney-aging axes. Publishing data provided a novel avenue for the diagnosis of bowel disease and kidney disease by host- and microbial-derived tryptophan metabolites and the discovery of therapeutic agents for treatment of patients with bowel disease and kidney disease by regulating the tryptophan metabolic enzymes. Tryptophan metabolism has a critical role in physiology and physiopathology. The main pathways are differentially affected in multiple diseases but remain tightly interconnected and interacted. The rapidly expanding knowledge on key roles of tryptophan metabolism in multiple diseases such as ageing-related diseases, bowel diseases and kidney diseases has illuminated promising therapeutic targets. Tryptophan metabolism has demonstrated to be as a regulator in age-related pathologies and lifespan in yeast, worms, flies, and mice. The use of small model organisms provides powerful tools to determine the enzymes and metabolites of tryptophan metabolites in the different stages of life from birth to old age. These models play a key role in reveal receptors and pathways that induce the effects of aberrant tryptophan metabolism. Although drug research focuses mainly on the development of IDO1 and TDO inhibitors, there are clearly novel targets and indications rapidly discovery, such as the development of kynurenine monooxygenase inhibitors. Future efforts should be implemented state-of-the-art analytical tools to evaluate tryptophan metabolism in a tissue-specific approach. In addition, it is necessary to further identify the relevance of intermediate metabolites and related-enzymes in kynurenine pathway. Moreover, future observation and clinical trials will further shed light on the druggability of the kynurenine pathway.

Extensive studies have shown that bowel disease and renal injury led to intestinal epithelial cell barrier damage and the dysbiosis of gut microbiota. In contrast, damaged intestinal epithelial cell barrier and microbial dysbiosis exacerbated bowel disease and renal injury. The microbial-derived uremic toxins enter bloodstream circulation through damaged intestinal epithelial cell barrier and mediate endothelial cell injury by producing local or systemic low-level inflammation that exacerbate bowel disease and renal injury. Increasing publications have demonstrated that ageing was implicated in renal injury 129. Ageing aggravates bowel disease and kidney disease. Intestinal tryptophan metabolism was directly or indirectly controlled by microbiota. Therefore, tryptophan metabolism in gut, as an actionable actor, exhibited a therapeutic perspective, through either molecule targeting a specific pathway or utilizing microorganisms manipulating tryptophan metabolism. Current achievements may extend our understanding of host-microbial crosstalk in various diseases. Although identified many bacteria could metabolize tryptophan, the largely mediators in human gut remain unknown even now the large amount of fecal metagenomic data available. When we continue to determine the mediators, the aims should focus on combining microbiome profiles with quantifying tryptophan metabolites using metagenomics-metabolomics approach in human fecal samples. This will allow us to reveal important relationship between gut microbiota and tryptophan metabolites, which can be validated by using *in vitro* experiments and metabolic phenotyping. Once we identify tryptophan-related microorganisms in gut of the different age stages such as infants, adolescent, adults and elderly, we must decipher the accurate role of individual metabolites in host pathophysiology and reveal their precise molecular mechanisms in the different intestinal segments and specific tissues by animal models and human interventions. In addition, the receptors should be identified to recognize specific metabolites. Furthermore, AHR, as the receptors of tryptophan-derived metabolites, underline the interaction importance between tryptophan metabolites and AHR in human cells and not solely in murine models in the different mammals. Currently, the connections between tryptophan metabolites and human diseases remain rather tentative and most findings from animal models. The interaction complexity of host-microbiota and the sophistication of the diseases and models demand further to clarify targets and intervention effects. In addition, an extensive understanding of the dynamics of tryptophan metabolites and their underlying function in the different stages of life from birth to old age, from health to disease is needed. Taken together, the improvement of dysregulated tryptophan metabolism using pharmacological inhibitors or endogenous metabolites will provide new therapeutic strategy for preventing and treating CKD. This review presents concept-driven diagnostic and therapeutic strategies for the management of patients with CKD by gut-kidney-aging axes.

## Funding

This study was supported by National Natural Science Foundation of China (Nos. 82474062, 82274192 and 82274079) and Shaanxi Key Science and Technology Plan Project (No. 2019ZDLSF04-04-02).

## Figures and Tables

**Table 1:** Tryptophan metabolites by host and gut microbiota and their mechanisms and biological effects in health and diseases

Metabolites (Origin)	Microbial phyla/species/cells	Molecular pathway/targets	Biological effects	References
Indole (microbiota)	*Bacteroides species* (*B. thetaiotaomicron; B. ovatus*; *B. fragilis*); *Clostridium species (C. limosum*; *C. bifermentans*; *C. malenomenatum*;* C. tetani*;* C. ghoni*; *C. lentoputrescens*; *C. tetanomorphum*; *C. sordellii*; *C. bartlettii*); *Desulfovibrio vulgaris*; *Enterococcus faecalis*; *Escherichia coli*; *Fusobacterium nucleatum*; *Haemophilus influenza*; *Peptostreptococcus asscharolyticus*; *Lactobacillus Bifidobacterium longum*; *Parabacteroides distasonis*; *Eubacterium hallii*.	AHR ligand; activate AHR; stimulate glucagon-like peptide-1 secretion.	Promote mucus; enhance IEC barrier function; increase portal blood pressure and decrease arterial blood pressure.	130, 131
IAA (microbiota)	*Bacteroides species* (*B. thetaiotaomicron*; *B. eggerthii*; *B. ovatus*; *B. fragilis*); *Bifidobacterium species* (*B. adolescentis*, *B. longum subsp. Longum*;* B. pseudolongum*); *Clostridium species* (*C. bartlettii*; *C. difficile*; *C. lituseburense; C. paraputrificum*; *C. perfringens*; *C. putrefaciens*; *C. saccharolyticum*; *C. sticklandii*; *C. subterminale*); *Eubacterium species* (*E. hallii*; *E. cylindroides*); *Escherichia coli*; *Parabacteroides distasonis*; *Peptostreptococcus asscharolyticus*.	AHR ligand; activate AHR, p38MAPK and NF-κB pathway.	Protect IEC barrier function; mediate inflammation; induce endothelial and vascular dysfunction; mediate renal fibrosis.	43, 132
3-methylindole /Skatole (microbiota)	*Clostridium species* (*C. bartlettii; C. scatologenes; C. drakei*); *Eubacterium species* (*E. cylindroides*; *E. rectal*); *Bacteroides thetaiotaomicron*; *Butyrivibrio fibrisolvens*; *Lactobacillus spp.*; *Megamonas hypermegale*; *Parabacteroides distasonis*.	AHR ligand; activate AHR pathway; inhibit p38 pathway and lipid peroxidation.	Induce IEC death; inhibit IEC apoptosis; pulmonary toxin.	43, 133, 134
IPA (microbiota)	*Clostridium species* (*C. botulinum*; *C. caloritolerans*; *C. paraputrificum*; *C. sporogenes*; *C. cadvareris*); *Peptostreptococcus species* (*P. asscharolyticus*; *P. russellii*; *P. anaerobius*; *P. stomatis*).	AHR ligand; activate AHR and pregnane X receptor pathways; inhibit β-amyloid fibril formation; reduce lipid peroxidation.	Antiinflammation; maintain IEC barrier function and mucosal homeostasis; treat Alzheimer's disease.	40, 41, 135
Indoleacrylic acid (microbiota)	*Peptostreptococcus species* (*P. russellii*;* P. anaerobius*;* P. stomatis*);* Clostridium sporogenes*.	AHR ligand; activate AHR; promote interleukin-10 secretion; inhibit TNF production.	Promote IEC barrier function; antiinflammation; retard IBD.	40, 41
IAld (microbiota)	*Lactobacillus species* (*L. acidophilus*; *L. murinus*;* L. reuteri*).	AHR ligand; activate AHR; promote interleukin-22 production.	Antiinflammation; maintain IEC barrier function; promote mucus; maintain homeostasis.	42, 136, 137
ILA (microbiota)	*Anaerostipes species* (*A. hadrus*; *A. caccae*); *Bacteroides species* (*B. thetaiotaomicron*; *B. eggerthii*; *B. ovatus*; *B. fragilis*); *Bifidobacterium species* (*B. adolescentis*; *B. bifidum*; *B. longum subsp. Infantis*; *B. longum subsp. Longum*; *B. pseudolongum*); *Clostridium species* (*C. bartlettii*; *C. perfringens*; *C. sporogenes*; *C. saccharolyticum*); *Eubacterium species* (*E. rectal*; *E. cylindroides*); *Lactobacillus species* (*L. murinus*; *L. paracasei*; *L. reuteri*); *Faecalibacterium prausnitzii*; *Escherichia coli*;* Megamonas hypermegale*; *Parabacteroides distasonis*; *Peptostreptococcus asscharolyticus*; PC12 cells; immature intestinal enterocytes.	AHR ligand; activate AHR; regulate Ras/ERK pathway; increase tyrosine protein kinase A receptor and CREB expression; reduce interleukin-8 expression.	Antiinflammation; potentiate nerve growth factor-induced neurite outgrowth.	40, 42, 43, 137-140
Tryptamine (microbiota)	*Clostridium sporogenes*; *Ruminococcus gnavus.*	AHR ligand; activate AHR; inhibit NF-κB and TNF-α expression.	Antiinflammation; inhibit liver inflammatory responses.	14, 141
IS (microbiota, host)	*Clostridium species* (*C. sporogenes*; *C. bartlettii*); *Escherichia coli*; *Lactobacillus Bifidobacterium longum*; *Bacteroides fragilis*; *Parabacteroides distasonis*; *Eubacterium hallii.*	AHR ligand; activate AHR and NF-κB pathways; regulate tissue factor.	Mediate oxidative stress and inflammation; induce vascular injury and thrombosis; mediate renal fibrosis.	132, 136, 142
Melatonin (microbiota, host)	*Clostridium sporogenes*; *Escherichia coli*.	Inhibit NF-κB pathway; suppress COX-2 levels.	Antiinflammation; maintain IEC barrier function.	143-145
Serotonin (microbiota, host)	*Clostridium sporogenes*; *Escherichia coli.*	Suppress COX-2 levels; regulate glucose homeostasis.	Antiinflammation; maintain IEC barrier function.	146, 147
Kynurenine (host)	Intestinal epithelial cells; Hk-2 cells.	AHR ligand; activate AHR; regulate interleukin-10 receptor.	Antiinflammation; Maintain IEC barrier function; mediate renal fibrosis.	116, 132, 148
5-MTP (host)	HK-2 cells; cardiomyocytes; fibroblasts; A549 cells; human umbilical vein endothelial cells.	Inhibit NF-κB and p38MAPK pathways; maintain Nrf2 pathway; retard EMT; reduce ROS.	Antiinflammation; retard renal fibrosis; protect heart IRI; suppress A549 migration and invasion.	33, 34, 149, 150

CREB, cyclic AMP (cAMP)-responsive element binding protein; ROS, reactive oxygen species; IRI, ischemia reperfusion injury; TrkA, tyrosine protein kinase A; NGF, nerve growth factor; PC12, rat adrenal pheochromocytoma cell line; IEC, intestinal epithelial cells; IBD, inflammatory bowel disease.

## Abbreviations

5-HT: 5-hydroxytryptamine
5-HTP: 5-hydroxytryptophan
5-MT: 5-methoxytryptamine
5-MTP: 5-methoxytryptophan
AKI: acute kidney injury
ArAT: aromatic amino acid aminotransferase
AHR: aryl hydrocarbon receptor
AA-NAT: arylal kylamine N-acetyltransferase
CRF: chronic renal failure
COX-2: cyclooxygenase-2
ESRD: end-stage renal disease
eGFR: estimated glomerular filtration rate
FOXO: fork head box
HIOMT: hydroxyindole-O-methyltransferase
IAA: indole-3-acetic acid
IAld: indole-3-aldehyde
ILA: indole-3-lactic acid
IPA: indole-3-propionic acid
IDO: indoleamine 2,3-dioxygenase
ILDH: indolelactic acid dehydrogenase
IS: indoxyl sulfate
IBD: inflammatory bowel disease
IκB: inhibitor of kappa B
IIS: insulin/insulin-like growth factor
IBS: irritable bowel syndrome
Keap1: kelch-like ECH-associated protein 1
mTOR: mammalian target of rapamycin
N-acetyl-5-HT: N-acetyl-5-hydroxytryptamine
NAD+: nicotinamide adenine dinucleotide
Nrf2: nuclear factor erythroid 2-related factor 2
NF-κB: nuclear factor kappa B
SERT: serotonin-selective reuptake transporter
TDO: tryptophan 2,3-dioxygenase
TPH1: tryptophan hydroxylase 1
TPH2: tryptophan hydroxylase 2
TnaA: tryptophanase
UUO: unilateral ureteral obstruction
